# Morphological and Functional Alterations of Alveolar Macrophages in a Murine Model of Chronic Inflammatory Lung Disease

**DOI:** 10.1007/s00408-015-9797-4

**Published:** 2015-08-30

**Authors:** Julia Désirée Boehme, Sabine Pietkiewicz, Inna Lavrik, Andreas Jeron, Dunja Bruder

**Affiliations:** Infection Immunology Group, Institute of Medical Microbiology and Hospital Hygiene, Infection Control and Prevention, Otto-von-Guericke University, Leipziger Straße 44, 39120 Magdeburg, Germany; Immune Regulation Group, Helmholtz Centre for Infection Research, Inhoffenstraße 7, 38124 Brunswick, Germany; Department of Translational Inflammation Research, Institute of Experimental Internal Medicine, Otto-von-Guericke University, Pfälzer Platz, Gebäude 28, 39106 Magdeburg, Germany

**Keywords:** Chronic lung disease, Inflammation, Alveolar macrophage, Macrophage polarization, Infection

## Abstract

**Purpose:**

Chronic lung inflammation commonly induces a multitude of structural and functional adaptations within the lung tissue and airspaces. Yet the impact of a persistent inflammatory environment on alveolar macrophages is still incompletely understood. Here, we examined morphology and function of alveolar macrophages in a transgenic mouse model of chronic lung disease.

**Methods:**

Imaging flow cytometry, flow cytometry, and microscopic evaluation of alveolar macrophages isolated from healthy and inflamed lungs were performed. Gene expression of polarization markers was compared by quantitative real-time RT-PCR. The pro-inflammatory immune response of alveolar macrophages toward bacterial ligands was assessed in in vivo clodronate-liposome depletion studies.

**Results:**

Chronic lung inflammation is associated with a substantially altered, activated alveolar macrophage morphology, and blunted TNF-*α* response by these cells following stimulation with ligands derived from the respiratory pathogen *Streptococcus pneumoniae*.

**Conclusions:**

These results demonstrate pleiotropic effects of pulmonary inflammation on alveolar macrophage phenotype and function and suggest a functional impairment of these cells during infection with airborne pathogens.

## Introduction

Chronic lung diseases (CLDs) are multi-factorial inflammatory disorders and frequently involve alterations of structural as well as hematopoietic cells in the airways. In line with this, substantial molecular alterations of alveolar macrophages (AMs) were described in asthma [1], chronic obstructive pulmonary disease (COPD) [2], and fibrosis [3] disease settings. Beyond their classical scavenger function, AMs are dedicated pulmonary sentinels and shape host defense via the initiation of a cytokine immune response following recognition of pathogen-associated molecular patterns (PAMPs). Notably, AMs (and other macrophage populations) have a unique propensity to dynamically adapt to the local microenvironment via polarization into M1 or M2 phenotypes, which are associated with unique physiological functions during inflammatory processes [4].

In recent years, it was shown by others that macrophage polarization states are intimately linked to a unique morphology [5] and that vice versa patterning of macrophages into a distinct cell shape induces their polarization [6]. However, these observations were either made in in vitro approaches and/or in a non-lung environment and a link between morphology and function for macrophages is still missing in respiratory inflammatory disorders.

In the present study, we aimed to elucidate the impact of a persistent inflammatory environment on alveolar macrophage phenotype and function in vivo. To this end, we utilized the SPC-HAxTCR-HA transgenic mouse model previously established and extensively characterized in our lab [7–9]. In these mice, the concomitant expression of a neo-self antigen (influenza A/PR8 hemagglutinin, HA) by alveolar epithelial cells and the generation of self-antigen specific (HA-specific) CD4^+^ T cells evokes the spontaneous development of chronic pulmonary inflammation. We have previously demonstrated that chronic lung inflammation in SPC-HAxTCR-HA mice is associated with multifocal interstitial, perivascular, and peribronchial lymphocytic infiltrations as well as the development of alveolar emphysema and hemosiderosis [7]. Here, we provide comparative morphological and functional analyses between alveolar macrophages of single transgenic SPC-HA (only pulmonary self-antigen expression; healthy control group) and double-transgenic SPC-HAxTCR-HA (pulmonary self-antigen expression and self-antigen specific T cells; chronic lung disease) mice.

## Materials and Methods

### Mice

TCR-HA [10], SPC-HA [7], and SPC-HAxTCR-HA [7] mice have been previously described. Mice were bred in the animal facility at the Helmholtz Centre for Infection Research in specific-pathogen-free (SPF) conditions and were used at 12–24 weeks of age. All animal experiments were performed according to national guidelines and were approved by government institutions (Niedersächsisches Landesamt für Verbraucherschutz und Lebensmittelsicherheit, file no. 33.14-42505-04-14/1715).

### Flow Cytometry and Imaging Flow Cytometry

Bronchoalveolar lavage (BAL) was performed by flushing the lungs once with 1 mL PBS and BAL cells were obtained by centrifugation. BAL cells were erythrocyte-depleted by treatment with ACK buffer (0.15 mM NH_4_Cl, 1 mM KHCO_3_, 0.1 mM EDTA, pH 7.2–7.4), Fc*γ*-receptors were blocked with anti-CD16/CD32 (clone: 93); cells were stained with anti-F4/80 (clone: BM8) and anti-CD11c (clone: N418, all BioLegend). In flow cytometry, single cells were analyzed using a LSR Fortessa flow cytometer (BD Biosciences). Alveolar macrophages (AMs) were identified as F4/80^+^ and—where indicated—CD11c^+^, autofluorescence^high^, forward scatter^high^, and side scatter^high^ cells. Autofluorescence signals were detected in FITC channel (500–550 nm). In imaging flow cytometry, alveolar macrophages were identified as F4/80^+^ and CD11c^+^. To exclude T cells from morphological analyses in imaging flow cytometry, cell suspensions were additionally stained with antibody against CD3 (clone: 145-2C11, eBioscience). Alveolar macrophages were analyzed by applying the image-based features intensity, area, and bright detail intensity on the SSC-channel. Analyses were conducted using a FlowSight System (×20 magnification, Amnis, part of EMD Millipore) Inspire acquisition software and IDEAS software version 6.0.

### Panoptic Staining with Pappenheim Method

For analyses of alveolar macrophage phenotype, BAL cells from SPC-HA and SPC-HAxTCR-HA mice were isolated and erythrocyte-depleted as described above. Cells were spun (800 rpm, 15 min, RT) onto SuperFrost^®^ glass slides (Thermo Scientific), air dried, and stained with May-Grünwald and Giemsa reagent (both Merck). Slides were washed and air dried, and samples were covered with Neo-Mount (Merck) reagent and sealed by glass cover slips. Images were captured at 63× oil immersion objective using an Axio Imager Z2 microscope equipped with AxioCam MRc5 and ZEN software (both Carl Zeiss Microscopy GmbH).

### In Vivo Experiments

For in vivo bacterial stimulation experiments, a pneumococcal serotype 4 strain (*S.pneumoniae* TIGR4, ATCC BAA-334 [11] ) was used. Pneumococci were grown overnight on Columbia blood agar plates (BD) at 37 °C and 5 % CO_2_, single colonies were resuspended and cultured in Todd-Hewitt broth & yeast extract to mid-logarithmic phase (OD_600nm_ 0.3–0.5), washed, and diluted in PBS to the desired concentration. CFUs were determined by growing serial dilutions of the inoculums on blood agar plates. Bacterial suspension was spun down (17,900×g), the supernatant was removed, and the bacterial pellet was resuspended in ice-cold ethanol (70 % v/v) and incubated at 4 °C for 1 h. The bacterial suspension was again spun down, and the pellet was resuspended in 1 mL sterile PBS/Glycerol solution (25 % v/v). Plating after ethanol treatment was carried out to verify effective bacterial killing. Ethanol-killed bacterial suspensions were stored at −70 °C until further use. Mice were anesthetized by intraperitoneal injection of ketamine/xylazine, and 20 µL of bacterial suspension—equivalent to 10^6^ CFU—was oropharyngeally administered. Mice were sacrificed at 4 h post-administration, and BAL was performed by flushing the lungs with 1 mL PBS. In order to deplete alveolar macrophages, 50 µL of clodronate-liposomes (=250 µg encapsulated clodronate, purchased from ClodLip BV) was oropharyngeally administered to mice 3 days before treatment with ethanol-killed pneumococcal suspensions.

### Purification of Alveolar Macrophages

BAL cells were erythrocyte-depleted and incubated in DMEM (Gibco, supplemented with 10 % fetal bovine serum and antibiotics) for 90 min at 37 °C, 5 % CO_2_ in bacteriological plastic petri dishes. Non-adherent cells were then removed by extensively rinsing the surface with PBS. To verify effective purification, adherent cells were detached by treatment with 0.05 % Trypsin/EDTA (Gibco), stained with antibodies against F4-80 and CD11c and analyzed by flow cytometry. For RNA analysis, adherent cells were directly lysed on the plastic surface by treatment with RLT buffer (Qiagen, supplemented with 1 % mercaptoethanol), and RNA extraction protocol was applied. RNA was extracted and purified using RNeasy Mini and RNase-free DNase kit (both Qiagen) according to the manufacturer’s instructions.

### Quantitative Real-Time RT-PCR

For gene expression analyses of purified alveolar macrophages, RNA was analyzed using the SensiFAST^TM^ SYBR^®^ No-ROX One-Step Kit (Bioline) according to the manufacturer’s instructions. Transcript levels were normalized to the housekeeping gene *Actb*. Following primer sequences were used: Actb, *5′*-CTTCTTTGCAGCTCCTTCGT-*3′* (forward) and *5′*-TCCTTCTGACCCATTCCCAC-*3′* (reverse); Msr1, *5′*-GGAATAAGAGGTATTCCAGGTG-*3′* (forward) and *5′*-TTTGTCCTTTAGGTCCAGGAG-*3′* (reverse); Fizz1, *5′*-ACGAGTAAGCACAGGCAGTT-*3′* (forward) and *5′*-TGCCAATCCAGCTAACTATCCC-*3′* (reverse).

### Enzyme-Linked Immunosorbent Assay (ELISA)

For detection of TNF-*α* in BAL fluid, the Mouse TNF-*α* ELISA MAX™ Standard kit (BioLegend) was used according to the manufacturer’s instructions.

### Statistical Analyses and Artwork

Statistical evaluation and figure design was performed using Graph Pad Prism Software. Unpaired, two-tailed Mann–Whitney-test was chosen to compare two groups. For comparison of multiple groups, one-way or two-way ANOVA followed by Bonferroni post-test was applied.

## Results

### Altered Fluorescence Characteristics and Cellular Texture of Alveolar Macrophages in CLD

**Fig. 1:**
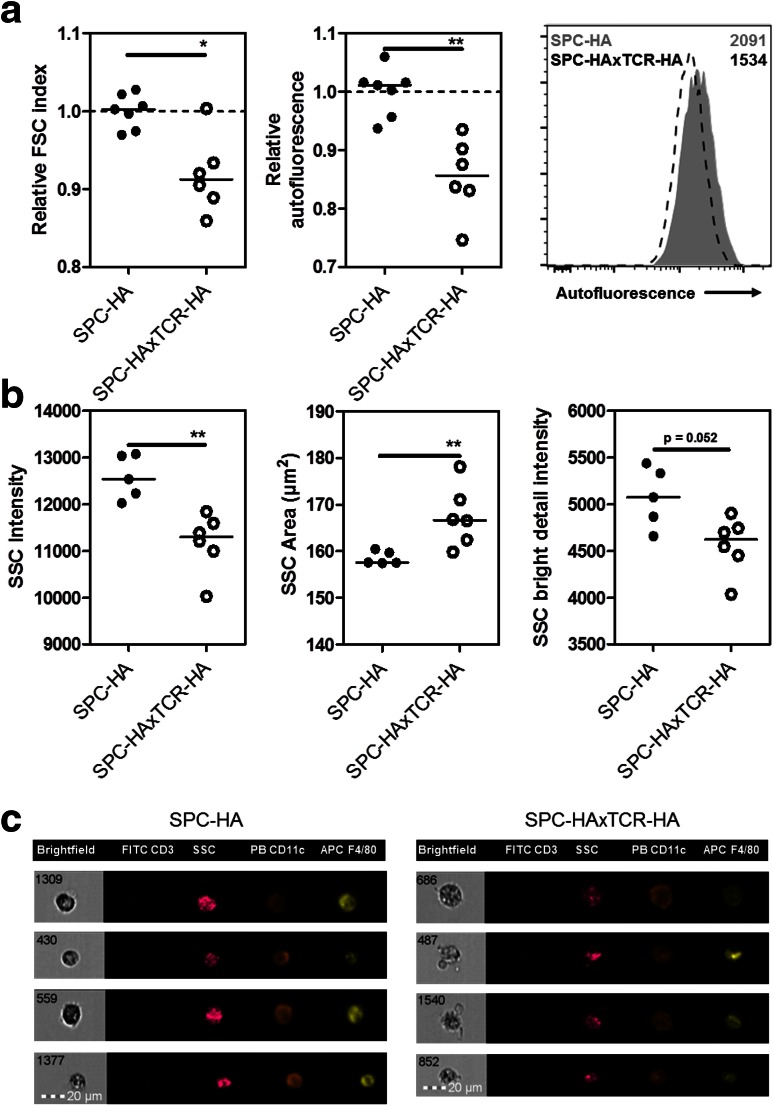
Flow cytometric analyses of AM morphology in healthy and diseased lungs. **a** BAL cells from SPC-HA (*n* = 6) and SPC-HAxTCR-HA (*n* = 6) mice were analyzed by FACS; AMs were identified as FSC^high^SSC^high^F4-80^+^autofluorescence^high^ cells. Relative FSC and autofluorescence were calculated by median fluorescence intensity (MFI) of individual sample/mean MFI of the SPC-HA (healthy) control group. Representative histogram depicts autofluorescent signal intensities of AMs from SPC-HA (*gray shaded*) versus SPC-HAxTCR-HA (*dashed line*) mice and their respective MFI (see numbers). **b** BAL cells from SPC-HA (*n* = 5) and SPC-HAxTCR-HA (*n* = 6) mice were analyzed by imaging flow cytometry (analyzed SSC-parameters: intensity, area, bright detail intensity); AMs were identified as F4-80^+^CD11c^+^CD3^−^ cells. Statistical analyses were performed by using unpaired, two-tailed Mann–Whitney-test. *p < 0.05, **p < 0.01 **c** Representative images from imaging flow cytometer FlowSight^®^ of alveolar macrophages from SPC-HA and SPC-HAxTCR-HA mice, *PB* pacific blue

For a primary characterization of the AM phenotype in chronic lung inflammation, these cells were recovered from SPC-HA and SPC-HAxTCR-HA mice by BAL and were subjected to morphologic analyses by conventional flow cytometry (FACS) as well as imaging flow cytometry. FACS analyses revealed AMs from diseased SPC-HAxTCR-HA mice to have significantly smaller size compared to AMs from healthy SPC-HA control mice—indicated by reduced forward scatter (FSC) properties. Moreover, quantification of autofluorescence—an intrinsic characteristic of alveolar macrophages—yielded significantly reduced indices for AMs from SPC-HAxTCR-HA mice (Fig. 1a). In imaging flow cytometry, these cells displayed altered cytoplasmic signals reflected by alterations of side scatter (SSC) characteristics (Fig. 1b, c), indicating a markedly changed cellular texture of alveolar macrophages in a persistent inflammatory pulmonary environment.

### Activated Phenotype of Alveolar Macrophages in Murine CLD

**Fig. 2:**
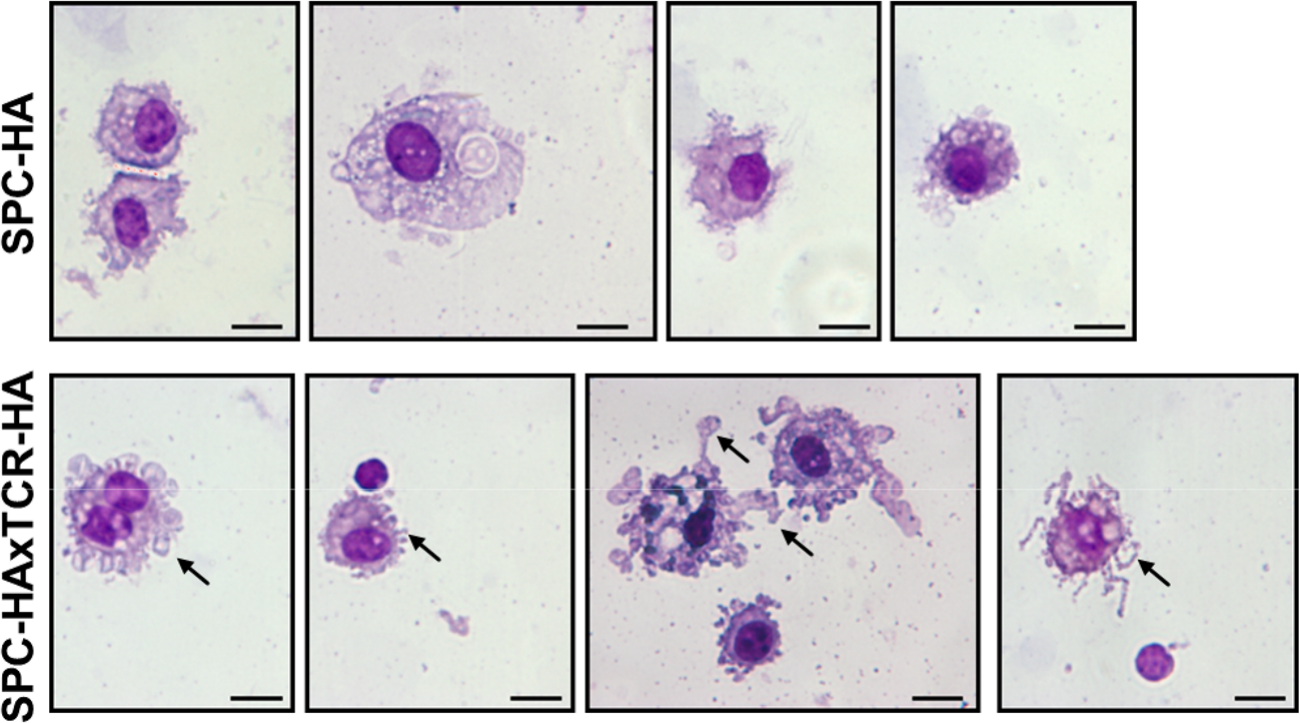
Analyses of AM morphology in healthy and diseased lungs by light microscopy. Cytospins from BAL samples from SPC-HA and SPC-HAxTCR-HA were stained with May-Grünwald and Giemsa reagent (Pappenheim method), and cell morphology was analyzed by light microscopy. Representative images of alveolar macrophages from *n* = 4 mice/group at ×63 magnification are depicted. *Scale bar* 10 µm. *Black arrows* indicate membrane protrusions

Further comparative morphologic analyses of alveolar macrophages were conducted using Pappenheim-stained cytospin samples of bronchoalveolar lavage fluid cells from SPC-HA and SPC-HAxTCR-HA mice. Microscopic evaluation demonstrated AMs from diseased SPC-HAxTCR-HA mice to display an activated phenotype, specified by increased formations of membrane protrusions (Fig. 2, see black arrows).

Together, the observed morphologic alterations provide first evidence for an impact of chronic pulmonary inflammation on alveolar macrophages and establish a basis for further investigations regarding functional alterations of AMs in murine lung disease.

### M2 Signature of Alveolar Macrophages in Murine CLD

**Fig. 3:**
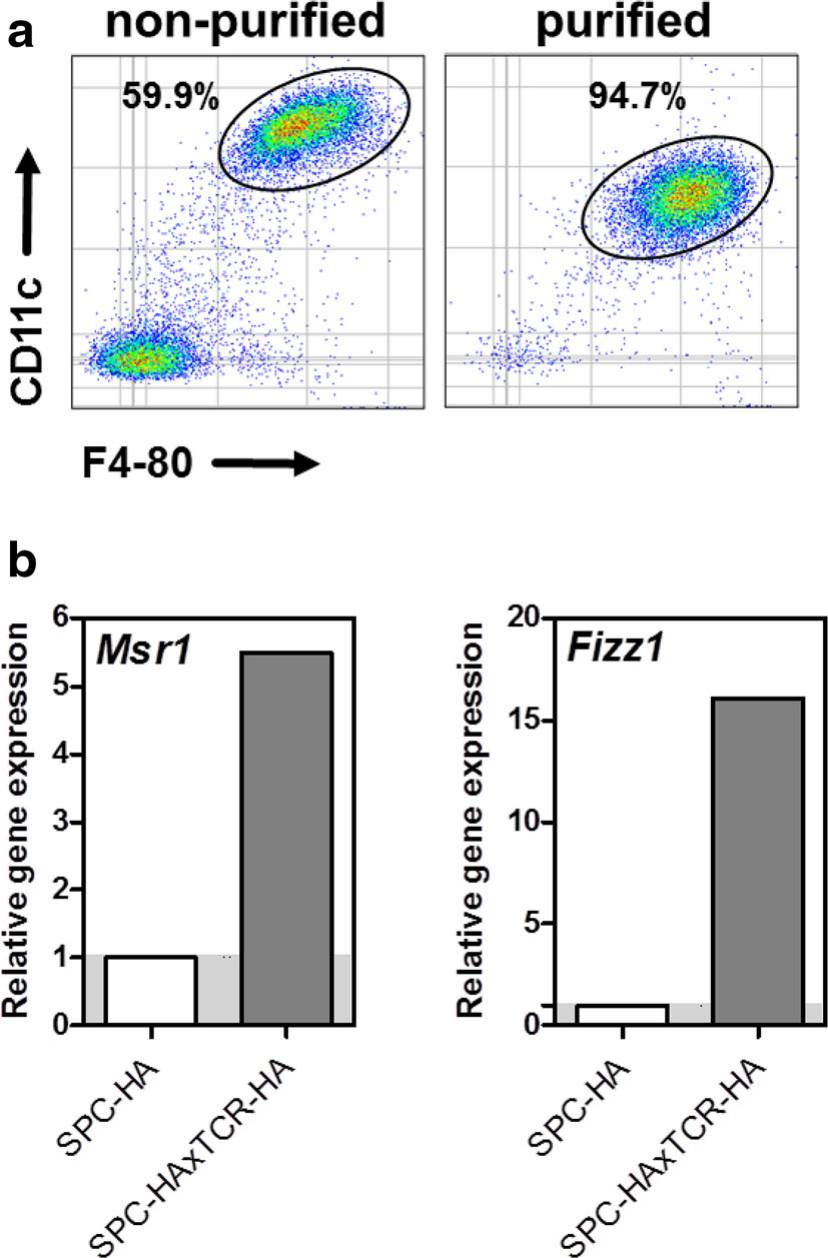
Analyses of purified AMs from healthy versus diseased lungs by quantitative real-time RT-PCR. **a** AMs from erythrocyte-depleted BAL cell suspensions were purified by adhesion (90 min, 37 °C) on bacteriological plastic, detached by trypsin–EDTA treatment, and purity was analyzed by FACS. *Left square* exemplarily depicts percentages of AMs (CD11c^high^, F4-80^high^) of analyzed singlets in non-purified BAL samples from SPC-HAxTCR-HA mice. *Right square* depicts AM percentages of BAL cells from SPC-HAxTCR-HA mice after purification. Note the decreased abundance of the surface molecule CD11c on AMs after trypsin–EDTA treatment. **b** Purified alveolar macrophages from SPC-HA and SPC-HA xTCR-HA mice were pooled (*n* = 3–5 mice/group), RNA was extracted, and relative expression of indicated transcripts was determined. Gene expression was normalized to the SPC-HA control group

Inflammatory conditions in multiple infectious as well as non-infectious disease settings are intimately linked to macrophage polarization into classically activated M1 or alternatively activated M2 phenotypes [12, 13]. In order to elucidate the impact of CLD on AM polarization status, BAL cells from SPC-HA and SPC-HAxTCR-HA mice were isolated and AMs were purified by adhesion. This method allowed for the fast and efficient isolation of highly pure (~95 %) alveolar macrophages (Fig. 3a). Comparative gene expression analyses of macrophage polarization markers were conducted by quantitative real-time RT-PCR. Here, up-regulation of the gene encoding for macrophage scavenger receptor 1 (*Msr1*, fold change 5.5) and Resistin-like molecule alpha1 (*Fizz1*, fold change 16.1), both molecules are known to be overrepresented in M2 macrophages, was observed (Fig. 3b).

### Alveolar Macrophages in Murine CLD are Hyporesponsive to Stimulation with Bacterial Ligands

**Fig. 4:**
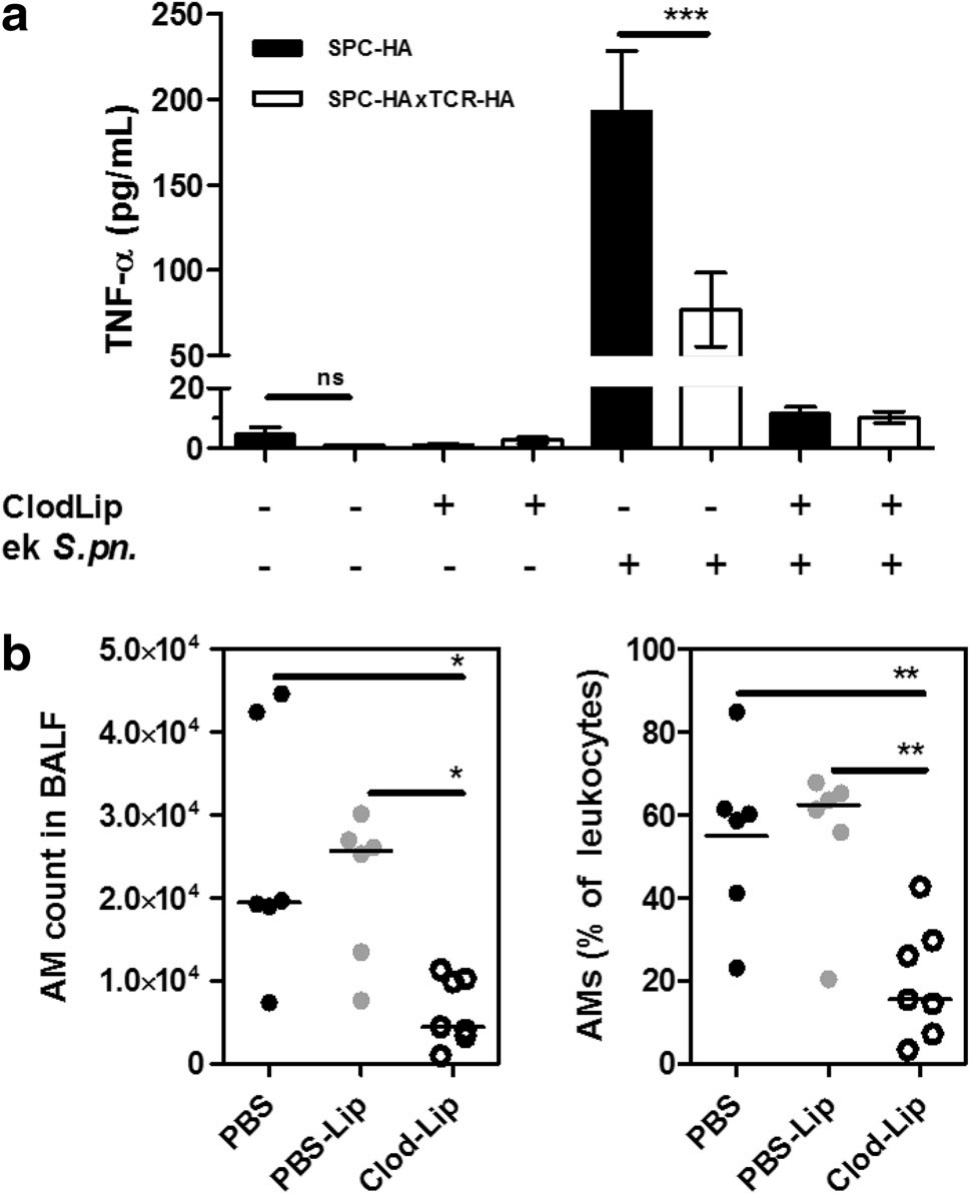
Quantification of the AM-dependent TNF-*α* response toward bacterial ligands in vivo. **a** Mice were oropharyngeally inoculated with 50 µL of clodronate-liposomes (ClodLip) on d0; control mice received PBS. At d3, mice were oropharyngeally administered with 10^6^ CFU ethanol-killed *S. pneumoniae* TIGR4 (ek *S.pn*.) or PBS, respectively. 4 h after bacterial inoculation, mice were sacrificed and TNF-*α* levels in BAL fluid were determined by ELISA. Depicted are mean results ± SEM of pooled samples from 2 independent experiments. (*n* = 6–10/group). Statistical significance was calculated using two-way ANOVA followed by Bonferroni post-test. ***p < 0.001. **b** SPC-HA mice were oropharyngeally inoculated with 50 µL Clodronate-Liposomes (Clod-Lip), PBS-Liposomes (PBS-Lip) or PBS only. At d3 post administration mice were sacrificed, and alveolar macrophages (CD11c^high^F4-80^+^autofluorescence^high^) in BAL fluid were quantified by flow cytometry. Data were pooled from two independent experiments with similar outcome. Statistical significance was calculated using one-way ANOVA followed by Bonferroni post-test. *p < 0.05; **p < 0.01

Following recognition of respiratory pathogens, alveolar macrophages mount a pro-inflammatory immune response by secreting (among other mediators) TNF-*α*. This step is critical for the rapid elimination of microbial threats but, on the other hand, involves the risk to develop immune pathology at the expense of lung tissue integrity. In order to test a possible influence of chronic lung inflammation on alveolar macrophage functionality, SPC-HAxTCR-HA mice and healthy controls were oropharyngeally inoculated with ethanol-killed *Streptococcus pneumoniae* TIGR4, and the early airway inflammatory response was quantified by assessing TNF-*α* levels in BAL fluid (BALF) at 4 h following administration. As expected, bacterial stimulation led to an early and robust cytokine response in the airways of healthy SPC-HA mice (Fig. 4a). Strikingly, TNF-*α* levels were ~60 % decreased in the diseased SPC-HAxTCR-HA group. In order to verify that this blunted inflammatory response was mediated by alveolar macrophages, these cells were depleted by oropharyngeal administration of clodronate-liposomes (ClodLip) 3 days prior bacterial stimulation. Clodronate treatment led to a 75 % reduction of viable AMs (Mean ± SEM AM counts PBS-group vs. ClodLip group: 25383 ± 6040 vs. 6330 ± 1536) within 3 days, whereas the single administration of control liposomes (PBS-Lip) or carrier solution (PBS) had no effect on alveolar macrophage numbers (Fig. 4b). Notably, TNF-*α* levels following bacterial stimulation in AM-depleted mice were substantially reduced and close to detection limit in both mouse groups. This validates AMs to be the main early airway TNF-*α* source following pneumococcal stimulation and, more importantly, suggests a functional impairment of AMs in chronic lung disease.

## Discussion

In the present study, we aimed to dissect the impact of chronic lung disease on the alveolar macrophage phenotype. To this end, we utilized the SPC-HAxTCR-HA transgenic mouse model for T cell-mediated lung inflammation. Despite different etiologies, a participation of lymphocyte immune responses has been suggested for the pathogenesis of several lung disorders such as COPD [14] or sarcoidosis [15] and SPC-HAxTCR-HA lungs phenocopy [7] histopathological manifestations of human CLD, e.g., airway remodeling and lymphocytic infiltrations.

By using fluorescence-based cytometric assays, we detected several important morphological changes of alveolar macrophages in our in vivo model. In detail, we show that their size, structural, and intrinsic fluorescence properties are significantly affected within an inflammatory environment. Interestingly, alterations in airway macrophage SSC and autofluorescence have also been reported in another study in SHIP1 (an inositol polyphosphate-5-phosphatase)-deficient mice, that spontaneously develop severe lung inflammation associated with a pathologic M2-macrophage phenotype [16]. These findings are of general interest for two reasons. First, FACS technology provides a valuable and commonly utilized tool to characterize inflammatory processes on airway mucosal surfaces. Yet the massive autofluorescence signals of airway macrophages within, e.g., FITC, PE, and PerCp detectors require well-considered panel design and the utilization of additional control stainings to correctly separate large intrinsic (false) from antibody-derived (true) fluorescence signals. Since we and others have proven fundamental differences of AM fluorescence properties in healthy versus pathologic conditions, it is likely to observe similar effects in other non-infectious and infectious respiratory disease settings. In practice, this would adversely affect accuracy of, e.g., cell surface molecule quantitation. Thus, for similar studies using comparative flow cytometric investigations on airway macrophages, the aforementioned effects should definitely be considered. Second, information on the mentioned parameters offers a simple additive tool to characterize the alveolar macrophage phenotype in more detail and to detect morphologic alterations that possibly hint to molecular and functional cellular adaptations. Accordingly, we could provide a link between affected fluorescent properties and activation, polarization, and functional alterations in alveolar macrophages in CLD. Here, the blunted AM-dependent TNF-*α* response toward Gram-positive bacterial ligands in SPC-HAxTCR-HA airways suggests a functional impairment of these cells during infection with airborne pathogens. Of note, TNF-*α* was shown to exert protective efficacy in several models of respiratory infection [17–20]. In detail, this cytokine acts on macrophages as well as neutrophils by enhancing phagocytosis of opsonized bacteria and antibody-dependent cytotoxicity [21, 22]. On the other hand, it was previously demonstrated that lung-specific overexpression of TNF-*α* in mice was linked to a disease phenotype mirroring hallmark features of emphysema and pulmonary fibrosis [23]. In this context, macrophages from pre-diseased human and murine lungs were found to mount blunted cytokine responses upon stimulation with bacterial ligands [24, 25]. It is speculated that this airway hyporesponsiveness provides an advantage to the host by evading PAMP-induced immunopathology that would further compromise tissue integrity in the pre-inflamed lung. However, given the two faces of TNF-*α* (and other pro-inflammatory molecules) in alveolar macrophage-mediated immune responses, further studies will be needed to explore their role on the shape of antibacterial host defense in the pre-diseased lung.

## Conclusion

Using a model of chronic lung disease, we provide a link between phenotypical changes in alveolar macrophages to distinct functional alterations. We further plan to characterize the alveolar macrophage phenotype in more detail and aim to elucidate the functional role of these adaptations on pulmonary immunity.
